# *Listeria monocytogenes* in Ready-to-Eat Seafood and Potential Hazards for the Consumers

**DOI:** 10.1155/2012/497635

**Published:** 2012-06-16

**Authors:** Patrizia Gambarin, Cristian Magnabosco, Marina Nadia Losio, Enrico Pavoni, Antonietta Gattuso, Giuseppe Arcangeli, Michela Favretti

**Affiliations:** ^1^Istituto Zooprofilattico Sperimentale delle Venezie, Viale dell'Univeristà 10, 35020 Legnaro, Italy; ^2^Istituto Zooprofilattico Sperimentale della Lombardia e dell'Emilia Romagna, Via Antonio Bianchi 7/9, 25124 Brescia, Italy; ^3^Dipartimento di Sanità Pubblica Veterinaria e Sicurezza Alimentare, Istituto Superiore di Sanità, Viale Regina Elena 299, 00161 Roma, Italy

## Abstract

The risk of exposure to *Listeria monocytogenes* (*L. monocytogenes*) when consuming Ready-to-Eat (RTE) seafood was assessed in the Veneto Region (Italy). Thirty-eight samples were analyzed, each sample consisted of three subunits belonging to the same batches. The first of the three units was examined immediately, the second was stored at +4°C (for all of its shelf-life) and the third at +10°C (for the latter third of its *shelf-life*) before the analysis. Chemical-physical and microbiological parameters were tested simultaneously. Culture results showed the presence of viable *L. monocytogenes* in 9 (23,68%) of the 38 samples analysed, 3 (33,33%) of which with a concentration >100 cfu/g. PCR tests yielded 12 *L. monocytogenes* positive samples. Semipreserves with aw (water activity) and pH values that favour *L. monocytogenes* growth were the only ones to result positive to microbiological and PCR tests. Temperature proved to be an important factor as it limits the growth of *L. monocytogenes*, including products with potentially high competitive microbial charges. Four different serotypes were recovered and ribotyping has helped to highlight the genomic variability of *L. monocytogenes* strains in food. This supports the hypothesis that *L. monocytogenes* continues to evolve genetically to the detriment of phenotypic conservation.

## 1. Introduction

*L. monocytogenes* is an intracellular pathogen which, especially if foodborne, may induce what is known as listeriosis. Once ingested, *L. monocytogenes* can penetrate the intestinal endothelial barrier, the placental or the hematoencephalic barrier [1, 2]. The groups at higher risk of contracting the disease are young people, the old, (over 65), pregnant women, and immune compromised people, the YOPI, an acronym coined by De Cesare et al. [3, 4]. In healthy subjects, *L. monocytogenes* can lead to episodes of gastroenteritis and fever [5, 6]. 

Listeriosis is considered a rare disease, its incidence in humans ranges between 0.1 and 11.3 cases/million [7], with a high mortality rate, up to 30% in the categories most at risk (YOPI) [8]. Based on an EFSA 2010 report, the incidence in Europe was of 3 cases/million of inhabitants [9]. Because the incubation period can span from 3 up to 60 days, this disease is often difficult to trace because it is not easy to isolate the food that is responsible for the infection. Europe has some well-documented episodes particularly in France [10], Finland [11], Switzerland [12], the UK [11], Belgium [13], and Ireland [14]. Distinct psychrotolerant characteristics allow *L. monocytogenes* to adapt to acidic conditions and to low water activity environments, making it an insidious threat to some kinds of food as ready-to-eat food (RTE) that is characterized by mild treatments and a medium-to-long *shelf-life*—a highly sought-after quality by today's consumer [15–22]. The fish products that pose potential risks include mainly cold smoked fish, raw carpaccio, and marinated fish. Smoked fish products in particular were reported to cause human infections [23–25]. In Europe, smoked salmon, more than all the other products, was reported to have surpassed the maximum threshold limits allowed for *L. monocytogenes* contamination [9].

The risk of consuming RTE seafood does not so much entail the contamination of the raw product, which will often have *L. monocytogenes*, but at low concentrations, as much as the product's characteristics which in time encourage its growth [26–28].

Inadequate consumer knowledge on how to store RTE food at home, at the right refrigerated temperature, has led to higher risks of *L. monocytogenes* growth [29, 30]. 

The goal of this study is to determine the distribution of *L. monocytogenes* in RTE fish semipreserves, as set out by EC Regulations 2073/2005 related to this category which distinguishes the foods that favour *L. monocytogenes* growth from those that do not. Different types of repfed products were examined, these include marinated seafood salads (with cephalopods, surimi, crustaceans, bivalves), marinated shrimps, cephalopods and salmon carpaccio, marinated mackerel, smoked herrings, and cold smoked salmon.

Packaged products marketed in the Veneto Region (Italy) were sampled to determine the levels of *L. monocytogenes. *The products inspected included ones with intrinsically favourable characteristics, thus ideal for *L. monocytogenes* growth, and those with unfavourable characteristics (*pH ≤ 4.4*, *a*_*w*_ ≤ 0.92; *pH ≤ 5.0* and *a*_*w*_ ≤ 0.94). Tests were carried on both these types of products stored, at 4°C and at 10°C, the latter being a more realistic simulation of household conditions which may experience thermal abuse. Each sample unit, aside from examining *L. monocytogenes* (qualitative and quantitative tests) also analysed the following: total aerobic mesophilic count, total psychrophiles count, total psychrophiles H_2_S producers, moulds, yeasts, and lactic bacteria to establish whether correlation exists in relation to the presence of *L. monocytogenes* Furthermore, enrichment broths were also tested for *L. monocytogenes* using PCR and serotyping and ribotyping of isolated strains and culture tests.

## 2. Materials and Methods

The samples, each consisting of 3 sample units coming from a production batch, upon reaching the laboratory, were stored in a refrigerator in their original package at a temperature of 4°C and 10°C until their *shelf-life* expiry (storage at 10°C in the last third of their *shelf-life* expiry). Product *shelf-life* ranged from 8 days (raw salmon carpaccio) to 70 days (seafood salads). During the collection phase at the various traders, only the samples which had not already surpassed half of their expiry period were considered. The products selected were both national and international products taken from 9 different stores. Upon reaching the laboratory, one sample unit was examined immediately, the other two were on the last day of expiry. In total 38 samples were analysed, amounting to 114 sample units. 

### 2.1. Microbial Analysis

- Total aerobic mesophilic count on agar plates with incubation in aerobiosis at 30°C for 72 hours (ISO 4833:2003).
- Total psychrotolerant count on iron agar plates (Lyngby) with incubation in aerobiosis at 15°C for 7 days.
- Total psychrophiles producing hydrogen sulphide count on iron plates (Lyngby) with incubation in aerobiosis at 15°C for 7 days.
- Count of the moulds and yeasts on Rose Bengal Chloramphenicol Agar plates with incubation in aerobiosis at 25°C for 5 days.
- Lactic bacteria count on MRS agar plates (final pH 6.4) with incubation in aerobiosis at 30°C for 72 hours.
- *L. monocytogenes *count on ALOA agar at 37°C for 48 hours (ISO 11290-2:1998/Amd 1 2004).
- Detection of *L. monocytogenes* on ALOA agar and PALCAM agar plates at 37°C for 48 hours following selective enrichment in Half Fraser and Fraser Broth (ISO 11290-1:1996/Amd 1 2004).

The quantitative and qualitative methods were carried out at the same time, the same day. 

### 2.2. Chemical and Physical Analysis

- pH measure using the Mettler Toledo MP 220 instrument, with temperature autocompensation.
- Water activity (*a*_*w*_) using Rotronic 29539 instrument (ISO 21807:2004).

### 2.3. Genomic DNA Extraction

Detection of *L. monocytogenes* consisted in taking 1 ml of enrichment broth (Half Fraser), after 24 hours of incubation, to extract DNA and carry out subsequent PCR tests for *Listeriaspp* and *L. monocytogenes. *DNA extraction was carried out on pellet, obtained after centrifugation of the enrichment broth (2,000 g for 2 minutes, followed by 12,000 g for 5 minutes on the surnatant). Once the surnatant was removed, PBS was added and 16,000 g underwent centrifugation for another 2 minutes. GenElute Bacterial Genomic DNA Mini Kit (Sigma) was used, following the manufacturer's instructions, protocol for gram positive bacteria.

### 2.4. Listerial Genus Detection by PCR

Nested PCR was the method used to target the codifying gene for 16S rRNA in which the amplified product of the first reaction becomes the template for subsequent nested reactions. The primers used in the first reaction were the forward primer LI1 5′-CTC CAT AAA GGT GAC CCT-3′ and the reverse primer U1 5′-CAG CMG CCG CGG TAA TWC-3′ [31]. The reaction took place in a final volume of 25 *μ*L with concentrations of 1X GeneAmp PCR Buffer II (Applied Biosystems), 1.5 mM of MgCl_2_, 0.2 mM of each dNTP, 0.2 *μ*M of both primers, 1.25 U of Ampli Taq DNA polymerase (Applied Biosystems), and 5 *μ*L of extracted DNA. For amplification, the thermal cyler GeneAmp PCR System 9700 (Applied Biosystem) was used with temperatures set at initial denaturation at 95°C for 3 min, followed by 25 cycles, each with a denaturation phase at 95°C for 90 sec, and an annealing phase at 50°C for 90 sec and an extension phase at 72°C for 2 min, followed by a final extension phase at 72°C for 10 min. The primers used for the nested reactions were forward primer LS1 5′-ACG ACC GCA ADG TTG AAA CT-3′ and reverse primer LS2 5′-GAC GTC ATC CCC ACC TTC CT-3′ manufactured at the Nucleic Acids Technology laboratory applied to foods at the Istituto Zooprofilattico Sperimentale of Brescia. The reaction was prepared in a final volume of 25 *μ*L with concentrations of 1X GeneAmp PCR Buffer II (Applied Biosystems), 1.5 mM of MgCl_2_, 0.2 mM of each dNTP, 0.2 *μ*M of both primer, with 0.75 U of Ampli Taq DNA polymerase (Applied Biosystems) and 2.5 *μ*L of extracted DNA. For amplification the thermal cycler GeneAmp PCR System 9700 (Applied Biosystem) was used with temperatures for initial denaturation set at 95°C for 3 min, followed by 35 cycles, each comprising a denaturation phase at 95°C for 30 sec, an annealing phase at 59°C for 30 sec, an extension phase at 72°C for 30 sec, followed by a final extension phase at 72°C for 5 min. Analysis of foreseen amplification, of 301 bp, was carried out after electrophoresis in agarose gel at 2.5% stained with ethidium bromide (final concentration on gel: 0.5 *μ*g/mL), by means of exposure to UV radiation.

### 2.5. *L. monocytogenes* Detection by PCR

A method to detect the HLY gene target (*haemolytic secreted pathogenic factor or hemolysin*) was used with forward primer LIS1 5′-CGG AGG TTC CGC AAA AGA TG-3′ and reverse primer LIS2 5′-CCT CCA GAG TGA TCG ATG TT-3′ [32]. Reaction was prepared using an end volume of 25 *μ*L with concentrations of 1X GeneAmp PCR Buffer II (Applied Biosystems), 1.5 mM of MgCl_2_, 0.2 mM for each dNTP, 0.2 *μ*M of both primers, 0.75 U of Ampli Taq DNA polymerase (Applied Biosystems) and 5 *μ*L of extracted DNA. Amplification was carried out in the thermal cycler GeneAmp PCR System 9700 (Applied Biosystem) with a temperature profile for initial denaturation set at 95°C for 3 min, followed by 35 cycles, each comprising a denaturation phase at 95°C for 30 sec, an annealing phase at 58°C for 30 sec and an extension phase at 72°C for 30 sec, followed by a final extension phase at 72°C for 5 min. Analysis of foreseen amplification, of 234 bp, was then conducted after electrophoresis in agarose gel at 2.5% stained with ethidium bromide (final concentration on gel: 0,5 *μ*g/mL), by means of exposure to UV radiation.

### 2.6. *L. monocytogenes* Serotyping by Multiplex PCR (M-PCR)

Serotyping by M-PCR was performed using primers as described by Doumith et al.[33]. Primers enable the identification of *Listeria *and the subdivision of strains belonging only to the *L. monocytogenes* species into four distinct serogroups. 

Serogroup 1 comprises serotype 1/2a and 3a; serogroup 2 of serotypes 1/2c and 3c; serogroup 3 serotypes 1/2b and 3b; and serogroup 4, serotypes 4b, 4d and 4e. 

The PCR mix included PCR Master Mix 1X (Qiagen, Milan, Italy), mix of primers (lmo0737, ORF2819, ORF2110, lmo1118 and prs), sterile distilled penta-H_2_O and the DNA extracted. PCR reaction conditions included an initial step of denaturation at 94°C for 3 minutes, 35 cycles at 94°C for 0.40 minutes, 53°C for 1.15 minutes, 72°C for 1.15 minutes and a final step at 72°C for 7 minutes.

PCR products underwent 2% agarose gel electrophoretic separation at 90 V for 90 minutes. Then, after being stained with ethidium bromide (10 ng/ml), they were visualised on UV transilluminator. 

### 2.7. *L. monocytogenes* Serotyping with Seroagglutination

Serotyping with antisera yielded 12 different serotypes of *L. monocytogenes* in terms of cellular surface, and the somatic “O” and flagellar “H” antigens. 

Seroagglutination was carried out to confirm the serotypes and serogroups obtained using the molecular method. The “SEIKEN” Listeria Antisera Kit (Denka Seiken co. LTD, Tokyo, Japan) was used according to a modified method outlined by Seeliger and Hohne [34].

### 2.8. *L. monocytogenes* Ribotyping

Positive *L. monocytogenes* colonies were detected and characterized using the Du Pont Qualicon Ltd. RiboPrinter Q system [3, 4]. The colonies were taken from agar plates using a plastic stick. They were suspended in a sample buffer. The solution was lysed at 85°C for 20 minutes, and two specific lysing agents were added. Subsequently, a batch containing eight containers to hold eight samples was inserted in the automated ribotyping instrument. In brief, RiboPrinter generates an enzymatic digestion of the solutions using the *Eco*RI restriction enzyme, and electrophoresis of DNA fragments was transferred onto a membrane. The membrane was hybridized using a specific chemiluminescent probe. Finally, the instrument detects the signal emitted by a CCD camera and software converts the images in ribotyping patterns. Ribotyping (of the species) is an automated process if more than 85% of the sample patterns resemble the reference patterns of the instrument's database. The latter were obtained from various international collections (e.g., ATCC, DSMZ, or JMC) and identified with a DUP-ID code (Dupont Identification). Genotypic characterization of bacterial strains consisted of comparing the strains of a batch, assigning to them what is called a ribogroup code (RG). Each ribotyped strain was compared with all the patterns of the profiles contained in the database. In general, if there was a similarity ≥93% between the profile of the strain investigated and that of the databank, the strain was assigned to the corresponding ribogroup of that profile. Ribogroup attribution is only possible if strains undergo simultaneous analysis or if analysis is conducted a few days later since the database has to be updated on an ongoing basis, accessing international databanks available on the internet. 

To obtain phylogenetic trees the results were extracted from a Pearson correlation (UPGMA method) with BioNumerics software version 6.1. 

## 3. Results 

The analyses were carried out on a range of 38 different commercial products 28 (73,68%) of which had *a*_*w*_ and pH values favourable for growth of *L. monocytogenes*, while 10 (26,32%) had unfavourable values instead (Table 1).

 Of the 28 samples with favourable characteristics for growth of the pathogen conditions, nine were positive for *L. monocytogenes* (32,14%), according to the standard culture methods (ISO 11290-1:1996/Amd 1 2004); of these, 3 (10,71%) had values exceeding the limit (100 cfu/g) established by EC Reg. no. 2073/2005. No sample with unfavourable characteristics was found positive for viable *L. monocytogenes* in microbiological testing.

Table 2 illustrates the corresponding values of *L. monocytogenes* qualitative test, quantitative test, and PCR results. *Listerial* genus was present in all 38 samples analysed according to the amplification results of the genus specific PCR reaction. Nonetheless, the species specific PCR demonstrated the presence of *L. monocytogenes* after enrichment in 12 samples only, of which 9 were also found to contain the pathogen by classical culture tests. The microbiological parameters are reported in Table 3. In general they rose when thermal abuse at 10°C occurred. In the 3 samples with a charge of >100 cfu/g, *L.* *monocytogenes* grew despite the presence of competitive flora, both lactic and alternating. Despite the increase of microbial values at the end of *shelf-life*, there were no significant changes in the product pH and *a*_*w*_ values. In evidence that of three samples with a higher than 100 cfu/g value (sample no. 2, 8 and 10), two were analysed after thermal abuse at 10°C (last third of its *shelf-life*), and one proved unfit at the time it was being taken. It is worth noting that the container of sample no. 2, at the end of its *shelf-life*, had bulging but no unpleasant odours. Other samples, when opened, had no significant organoleptic alterations.

**Figure 1:**
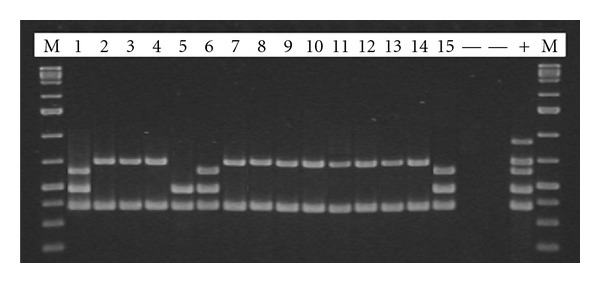
Multiplex PCR on isolated *L. monocytogenes* strains. M: marker; line 1: sample no. 1; lines 2-3-4: sample no. 7; line 5: sample no. 3; line 6: sample no. 5; lines 7-8-9: sample no. 2; lines 10-11: sample no. 8; lines 12-13: sample no. 9; line 14: sample no. 12; line 15: sample no. 10.

Serotyping of 15 isolated *L. monocytogenes* strains coming from RTE fish semipreserves was carried out using Multiplex PCR to separate the 4 main serotypes (1/2a, 1/2b, 1/2c and 4b) into 4 distinct groups. Confirmation of the serotype of single serogroups was achieved with the seroagglutination method (Table 4 and Figure 1).

The 15 strains of *L. monocytogenes* analysed came from 4 different serotypes: the highest percentage was related to serotype 1/2a (73,33%), followed by serotype 4b (13,33%), 1/2b (6,67%) and 4d (6,67%).

Ribotyping of *L.monocytogenes* colonies in microbiological tests (see Table 4) has yielded the broad variation of isolated strains, identifying 15 particular strains on the basis of species. The most diffuse type was DUP-1042, found in 9 samples (60%). DUP-20243, DUP-1043, and -DUP1052 were characterised in the strains examined, in 3 (20%), 2 (13.33%) and 1 (6.66%) sample, respectively.

**Figure 2:**
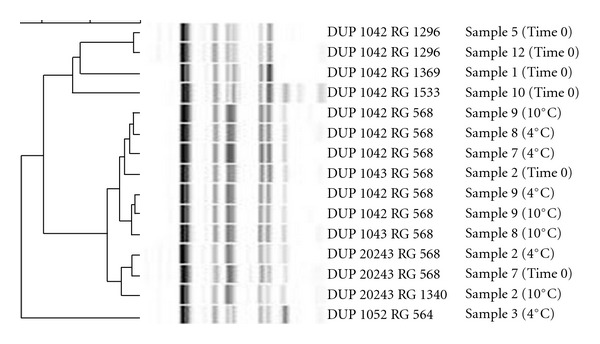
Phylogenetic distances between the various isolated *L.* *monocytogenes* strains.

In terms of ribogroup distribution, RG-568 was the most diffuse, found in 9 of the 15 samples (60%), and the other ribogroups were each found in only one sample, except RG-1296 which was found in 2 samples. Data from phylogenetic analysis on the restriction pattern (Figure 2) yielded a classification of three main strain groups identified with ribogroup 568, being highly phylogenetically related, in turn distant, though still phylogenetically related to the 4 strains grouped in the upper part of the dendrogram. The third and last group comprises of only one sample, no. 3. It was analysed after a *shelf-life* at a temperature of 4°C being very different from rest of the *L. monocytogenes* that were characterized. 

## 4. Discussion

The data demonstrate that there is a rather low probability of *L.monocytogenes* exceeding the 100 cfu/g limit in the RTE seafood distributed in the Veneto Region, which would at any rate be associated with improper storage of the product or with thermal abuse. In fact, of the 3 positive samples out of the 38, 2 had been stored at 10°C in the last third period of their lifecycle and only one was defective at the time it was collected. 

Analysis conducted at the end of *shelf-life* provides additional confirmation of the above-mentioned data.

The total charge of psychrophiles and of lactic bacteria found in the three positive samples was high, differently from Jameson's theory which affirms that the lactic charge should develop a sort of microbial competition against *L. monocytogenes* [35].

It is worth noting that 9 semi-preserves resulted positive at the qualitative microbiological test for *L. monocytogenes* (found in 25 g).

If at the end of *shelf-life* the above mentioned 9 samples had a <5 cfu level, the producer would still have to demonstrate, with appropriate studies and challenge tests, that *L. monocytogenes* will not grow and multiply. 

Our report reiterates that smoked and fresh salmon are particularly hazardous products.

In case of marinated products, just three samples had viable *L. monocytogenes.* At the end of *shelf-life* however no *L. monocytogenes* growth was detected. Differently from salmon, these products have much lower *a*_*w*_ and pH values, a factor that no doubt affects the growth of *L. monocytogenes* [36]. Moreover, smoked salmon production will have *L. monocytogenes* in the raw ingredient which cannot be eliminated in the phases leading to final packaging but can only be contained [37]. Seafood salads instead, since they are made with precooked raw ingredients, do not have *L. monocytogenes*. If there is any *L. monocytogenes* present in the end product, it implies that contamination occurred after the processing phases, after marinating.

The higher number of positive samples in the molecular biology tests and the inability to isolate culture underscore the presence of viable not culturable (VNC) organisms or no longer viable cells [38].

The PCR test compared to the culture is greatly targeted and more sensitive. Used on first enrichment broth it may prove useful in routine laboratory work because at least in PCR-negative cases it does not require microbiological tests.

The samples examined, all being positive to PCR tests for *Listeria*, confirm that these kinds of products are often contaminated with *Listeria*, but that such condition rarely evolves into hazardous *L. monocytogenes* concentrations.

In all the samples with unfavourable *a*_*w*_ and pH values for *L. monocytogenes* growth, the PCR test for *L. monocytogenes* was negative, confirming that even if the product was contaminated in the production phase, *L. monocytogenes* would be overwhelmed by the product's unfavourable environment.

The M-PCR method adopted was rapid, reproducible, and cost-effective. It can therefore be used in any equipped laboratory to perform molecular analysis. Its use however is not suggested to identify rare serotypes. For a serotype-specific analysis of isolated strains the traditional sero-agglutination method is recommended. The latter is no doubt less reproducible, more expensive, and requires specially trained staff, but it enables the detection of rare serotypes which in the years to come may be more frequently associated to cases of listeriosis. 

It is striking that 10 of the 11 *L. monocytogenes* strains isolated from salmon belonged to serotype 1/2a, as already confirmed by other scientific sources [39–41].

The agglutination method to confirm the serotypes of the single serogroups detected with M-PCR has highlighted a rare strain among the isolates belonging to the 4d serotype. 

Rare serotypes have been linked to an epidemic outbreak, as confirmed in a Finnish study by Maijala et al. in 2000 [11]. An epidemic case of listeriosis caused by the strain *L. monocytogenes* serotype 3a, isolated in packaged butter, was described therein. The epidemic outbreak affected 25 people, causing 6 deaths. 

Figure 2 shows the variability of strains found in the environment underscoring the need to develop methods that can assess the characterisation of pathogenicity, since same strains may correspond to different levels of pathogenicity.

Literature, in fact, documents *L. monocytogenes* and considers it a ubiquitous microorganism capable of adapting to different environmental conditions [42]. However, despite the various reports on genotyping and characterization [43, 44], there is still very little empirical evidence on the correlation between its presence in food and the ensuing pathologies its consumption generates in humans. This ought to encourage a more ample use of molecular characterization to provide information, even at the epidemiological level, on the distribution of *L. monocytogenes* in the environment, which though it maintains phenotypic homogeneity, undergoes ongoing phenomena of clonal evolution with small, yet significant, genome variations.

In addition, it is interesting to note that the 4 strains grouped in the upper part of the dendrogram identified as RG 1296, RG 1369, and RG 1533 were all isolated in samples analysed at time 0. While ribogroups 564, 568, and 1340 were instead isolated in samples analysed both at the beginning as well as at the end of the *shelf-life* period, this suggests that the latter may be more resistant in time to different storage conditions.

Because of the relatively small number of tested samples, this work should be considered as a preliminary study. Results should be confirmed throught further studies on different kind of products and increasing the number of samples.

## 5. Conclusions

Temperature plays a key role in preventing the growth of *L. monocytogenes.* In fact, the refrigeration chain is not always respected especially in case of products with a few weeks of *shelf-life*, as the products in this report, which may be subjected to temperature changes which do not bring about any evident organoleptic alterations. Production companies must therefore bear this in mind when certifying their products, using challenge tests to establish how safe a product is in terms of *L. monocytogenes*. They must also foresee temperature tests, in particular for products such as smoked salmon, traditionally from Northern European countries having lower average temperatures than countries like Italy also having less difficulty in managing the cold chain.

Products with *a*_*w*_ and pH characteristics that favour *L. monocytogenes* growth were the only ones to result positive to microbiological and PCR tests in this work, in support of the potential hazard of these products.

The highest percentage related to serotype 1/2a confirm similar investigations in seafood products and the genetic characterization using ribotyping demonstrates the genomic variability of *L. monocytogenes* strains also in this kind of food. This distinctive features could be interesting related to *L. monocytogenes* survival capacity under different storage conditions.

Nearly 30 years have gone by since Canadians Schelch [45] demonstrated that *L.* *monocytogenes* can infect human via contaminated food. The significant number of studies conducted since then has provided much data on this pathogen. Currently a lot is known about pathogenesis in humans and about its ability to resist and grow in different types of food.

Rightfully defined as an “*evolving pathogen*” by Bryan [46], it changes its phenotypic and genotypic characteristics under outside-induced stress conditions, with acidic substances, new additives, and with the innovative food technologies applied by food industries. The latter, when producing all the latest food products, have to constantly monitor them for this versatile pathogen. In tandem education campaigns geared at the consumers who may be exposed to *L. monocytogenes* must provide general rules and guidelines for proper food storage and for food preparation in the domestic environment.

## Figures and Tables

**Table 1:** Types of ready-to-eat seafood, country of origin.

Type of product	No. of samples with characteristics favourable to *L. monocytogenes *	No. of samples with characteristics unfavourable to *L. monocytogenes *	Country of origin
Cold smoked salmon	12	2	Other EU country
Marinated seafood salad	5	3	Italy
Shrimps in brine	3	3	Other EU country
Salmon Carpaccio	4	—	Italy
Octopus Carpaccio	2	—	Italy
Marinated cuttlefish	—	1	Italy
Cooked marinated mackerel	2	—	Italy
Cold smoked herrings	—	1	Other EU country

Total	28	10	—

**Table 2:** Products resulting positive to *L. monocytogenes. *

Sample no.	Type of product	Temperature of storage	*L. monocytogenes*culture qualitative test	*L. monocytogenes*culture quantitative test (cfu/g)	*L.monocytogenes* PCR
1	Seafood salad	T0	Positive	<5	Positive
		T0	Positive	<5	Positive
2	Salmon carpaccio	4^°^C	Positive	<5	Positive
		10^°^C	Positive	**330**	Positive
3	Marinated seafood Cocktail	4^°^C	Positive	<5	Positive
		10^°^C	Neg.	<5	Positive
4	Octopus Carpaccio	4^°^C	Neg.	<5	Positive
5	Cooked and marinated mackerel fillets	T0	Positive	<5	Positive
		10^°^C	Neg.	<5	Positive
6	smoked salmon	4^°^C	Neg.	<5	Positive
		10^°^C	Neg.	<5	Positive
		T0	Positive	<5	Positive
7	Fresh salmon in protective atmosphere	4^°^C	Positive	<5	Positive
		10^°^C	Positive	<5	Positive
		T0	Neg.	<5	Positive
8	Smoked salmon	4^°^C	Positive	<5	Positive
		10^°^C	Positive	**140,000**	Positive
9	Fresh salmon in protective atmosphere	4^°^C	Positive	<5	Positive
		10^°^C	Positive	<5	Positive
10	Smoked salmon	T0	Positive	**6,600**	Positive
11	Salmon Carpaccio	10^°^C	Neg.	<5	Positive
12	Cooked and marinated mackerel fillets	T0	Positive	<5	Positive

**Table 3:** Detailed description of microbial charges, a_w_ and pH values of each *L. monocytogenes*-positive sample (microbiological and PCR tests). Values expressed in CFU/g.

Sample n.	Types of product	Temperature of storage	Mesophiles count	Psicrophiles count	Psicrophiles H2S count	Moulds count	Yeasts count	Lactic count	aw	pH
1	Seafood salad	T0^(∗)^	9 × 10^8^	6.6 × 10^5^	<1	<1 × 10^2^	<1 × 10^2^	2.4 × 10^8^	0.0680556	5:01
		T0	2.8 × 10^4^	1.4 × 10^4^	6	4 × 10^2^	9 × 10^2^	9.1 × 10^2^	0.0666667	6:01
2	Salmon carpaccio	4^°^C^(∗∗)^	6.5 × 10^5^	3.8 × 10^5^	3.5 × 10^2^	2 × 10^2^	2.4 × 10^3^	6.4 × 10^5^	0.0673611	6:01
		10 ^°^C^(∗∗∗)(∗∗∗∗)^	**1.9 × 10** ^**8**^	**1.3 × 10** ^**8**^	**4.7 × 10** ^**4**^	**9 × 10** ^**2**^	**1.7 × 10** ^**4**^	**1.5 × 10** ^**8**^	**0.0673611**	**6:01**
3	Marinated seafood cocktail	4 ^°^C	7.5 × 10^4^	4.5 × 10^4^	<1	< 1 × 10^2^	<1 × 10^2^	3.7 × 10^4^	0.0673611	5:01
4	Octopus carpaccio	10^°^C	5.5 × 10^7^	3 × 10^6^	<1	<1 × 10^2^	<1 × 10^2^	4.6 × 10^7^	0.0680556	4:09
5	Cooked and marinated Mackerel Fillets^(∗∗∗∗∗)^	4^°^C	4.2 × 10^6^	2.4 × 10^4^	<1	< 1 × 10^2^	<1 × 10^2^	7.3 × 10^6^	0.0680556	5:03
6	Smoked salmon	T0	7.8 × 10^4^	7.4 × 10^3^	<1	<1 × 10^2^	7 × 10^2^	3.6 × 10^2^	0.0638889	6:01
		10^°^C	7.1 × 10^7^	6.1 × 10^7^	1.1 × 10^7^	<1 × 10^2^	1.5 × 10^5^	4.4 × 10^2^	0.0652778	6:03
		4^°^C	1.6 × 10^5^	9.6 × 10^4^	6.9 × 10^3^	4.9 × 10^3^	<1 × 10^2^	2 × 10^3^	0.0673611	6:01
7	Fresh salmon in protective atmosphere	10^°^C	1.2 × 10^6^	2.2 × 10^6^	8 × 10^4^	6 × 10^2^	5.9 × 10^3^	6.1 × 10^3^	0.0680556	6:00
		T0	2.7 × 10^6^	2.2 × 10^6^	2 × 10^5^	<1 × 10^2^	3.6 × 10^3^	9.8 × 10^3^	0.0673611	6:00
8	Smoked salmon	T0	7.6 × 10^7^	4 × 10^7^	1.8 × 10^5^	<1 × 10^2^	3 × 10^2^	2.8 × 10^7^	0.0673611	5:08
		4^°^C	**4.2 × 10** ^**8**^	**1.5 × 10** ^**8**^	**1.2 × 10** ^**7**^	**<1 × 10** ^**2**^	**4.5 × 10** ^**4**^	**7.3 × 10** ^**7**^	**0.0673611**	**6:02**
9	Fresh salmon in protective atmosphere	10^°^C	3.6 × 10^6^	2.9 × 10^6^	5.2 × 10^3^	<1 × 10^2^	3.9 × 10^3^	5.1 × 10^3^	0.0673611	6:02
		4^°^C	6.7 × 10^6^	1.7 × 10^5^	1.4 × 10^3^	<1 × 10^2^	3 × 10^2^	4.1 × 10^6^	0.0673611	6:02
10	Smoked salmon	10^°^C	**1.4 × 10** ^**7**^	**1.4 × 10** ^**7**^	**<1**	**<1 × 10** ^**2**^	**<1 × 10** ^**2**^	**4.3 × 10** ^**6**^	**0.0666667**	**6:02**
11	Salmon carpaccio	T0	7.5 × 10^6^	3.2 × 10^6^	1.3 × 10^5^	1 × 10^3^	3.5 × 10^3^	1.7 × 10^5^	0.0673611	6:00
12	Cooked and marinated mackerel fillets ^(∗∗∗∗∗)^	10^°^C	4.1 × 10^7^	1.2 × 10^5^	<1	<1 × 10^2^	<1 × 10^2^	2.7 × 10^8^	0.0680556	5:03

Note. The lines with bold lettering refer to the 3 samples with a *L. monocytogenes* concentration of >100 cfu/g.

^(∗)^: zero time, analysis of one of the three sample units on the day they reached the laboratory.

^(∗∗)^: 4^°^C, analysis at the end of *shelf-life* at 4^°^C storage conditions.

^(∗∗∗)^: 10 ^°^C, analysis at the end of *shelf-life* at 10^°^C storage conditions in the last third before final expiration.

^(∗∗∗∗)^: packaging with bulging at the end of *shelf- life* with no unpleasant odour.

^(∗∗∗∗∗)^: samples from the same company but from different batches.

**Table 4:** Serotyping and ribotyping results on isolated *L. monocytogenes* strains.

Sample no.	Types of product	Temperature of storage	Serotype	Ribotype	Ribogroup
1	Seafood salad	T0	4b	DUP 1042	1369
		T0	1/2a	DUP 1043	568
2	Salmon Carpaccio	4^°^C	1/2a	DUP 20243	568
		10^°^C	1/2a	DUP 20243	1340
3	Marinated seafood cocktail	4^°^C	1/2b	DUP 1052	564
		10^°^C			
4	Octopus carpaccio	4 ^°^C			
5	Cooked and marinated mackerel fillets	T0	4b	DUP 1042	1296
		10^°^C			
6	Smoked salmon	4^°^C			
		10^°^C			
		T0	1/2a	DUP 20243	568
7	Fresh salmon in protective atmosphere	4 ^°^C	1/2a	DUP 1042	568
		10 ^°^C	1/2a	DUP 1042	568
		T0			
8	Smoked salmon	4^°^C	1/2a	DUP 1042	568
		10^°^C	1/2a	DUP 1043	568
9	Fresh salmon in protective atmosphere	4^°^C	1/2a	DUP 1042	568
		10^°^C	1/2a	DUP 1042	568
10	Smoked salmon	T0	4d	DUP 1042	1533
11	Salmon carpaccio	10^°^C			
12	Cooked and marinated mackerel fillets	T0	1/2a	DUP 1042	1296
